# DVT incidence and risk factors in critically ill patients with COVID-19

**DOI:** 10.1007/s11239-020-02181-w

**Published:** 2020-06-30

**Authors:** Shujing Chen, Dingyu Zhang, Tianqi Zheng, Yongfu Yu, Jinjun Jiang

**Affiliations:** 1 Department of Pulmonary and Critical Care Medicine, Zhongshan Hospital, Fudan University, Shanghai, 200032 China; 2 Department of Tuberculosis and Respiratory Disease, Jinyintan Hospital, Wuhan, China; 3 Department of Biostatistics, School of Public Health, and The Key Laboratory of Public Health Safety of Ministry of Education, Fudan University, Shanghai, China; 4 Department of Clinical Epidemiology, Aarhus University, Aarhus, Denmark

**Keywords:** Deep vein thrombosis, Hypoalbuminemia, D-dimer, SOFA score, Coronavirus

## Abstract

Few data are available on the incidence of deep vein thrombosis (DVT) in critically ill COVID-19 with thrombosis prophylaxis. This study retrospectively included 88 patients in the ICU with critically ill COVID-19 at Jinyintan Hospital in Wuhan, China. All patients underwent compression ultrasonography for identifying DVT. Firth logistic regression was used to examine the association of DVT with sex, age, hypoalbuminemia, D-dimer, and SOFA score. The median (interquartile range [IQR]) age and SOFA score of 88 patients were 63 (55–71) years old and 5 (4–6), respectively. Despite all patients receiving guideline-recommended low-molecular-weight heparin (LMWH) thromboprophylaxis, the incidence of DVT was 46% (95% CI 35–56%). Proximal DVT was recognized in 9% (95% CI 3–15%) of the patients, while 46% (95% CI 35–56%) of patients had distal DVT. All of the proximal DVT combined with distal DVT. Risk factors of DVT extension occurred in all distal DVT patients. As Padua score ≥ 4 or IMPROVE score ≥ 2, 53% and 46% of patients had DVT, respectively. Mortality was higher in patients with acute DVT (30%) compared with non-DVT (17%), but did not reach statistical significance. Hypoalbuminemia (odds ratio [OR], 0.17; 95% CI 0.06–0.05, *P* = 0.001), higher SOFA score (OR per IQR, 2.07; 95% CI 1.38–3.39, *P* = 0.001), and elevated D-dimer (OR per IQR, 1.04; 95% CI 1.03–1.84, *P* = 0.029) were significant DVT risk factors in multivariable analyses. High incidence of DVT was identified in patients with critically ill COVID-19, despite the use of guideline-recommended pharmacologic thromboprophylaxis. The presence of hypoalbuminemia, higher SOFA score, and elevated D-dimer were significantly independent risk factors of DVT. More effective VTE prevention and management strategies may need to be addressed.

## Highlights

- The incidence of DVT in patients with critically ill COVID-19 was 46% despite the use of guideline-recommended thromboprophylaxis.
- The presence of hypoalbuminemia, SOFA score, and elevated D-dimer were predictors of DVT.
- More effective VTE prevention strategies are necessary for patients with critically ill COVID-19.

## Introduction

The global pandemic of novel coronavirus disease 2019 (COVID-19) is still under rapid progression worldwide and causes thousands of deaths daily [1], since its first outbreak in Wuhan, China since December 2019 [2]. Critically ill COVID-19, especially complicated by bedridden, obesity and other infection, is considered a risk factor for venous thromboembolism (VTE), a combination of deep vein thrombosis (DVT) and pulmonary embolism (PE) [3]. The potential mechanism of VTE in COVID-19 is unclearly indientified but is contributed by Virchow's triad: hypercoagulability, stasis and endothelial injury. Timely diagnosis of VTE is anticipated to pose a significant challenge, resulting to underdiagnosis and complications of respiratiory failure, arrhythmia, organ dysfunction [4]. Furthermore, VTE may extend length of ICU stay and aggravate outcome in COVID-19 [5].

DVT is often undetected in the intensive care unit (ICU) patients and probably leads to fatal PE [6, 7]. To date, there are few studies investigating DVT incidence and risk factors specifically in patients with critically ill COVID-19 with guideline-recommended LMWH prophylaxis. Some researchers found the incidence of PE in ICU were 4–30% with different prophylaxis strategies [8–13]. More data are needed to make new strategies of DVT prophylaxis in critically ill COVID-19. As such, identification of DVT incidence and risk factors in critically ill COVID-19, especially in the use of guideline-recommended pharmacologic thromboprophylaxis, remains important. Our study’s purpose was to identify the incidence of DVT and the independent risk factors of DVT in critically ill COVID-19 patients.

## Methods

### Study design and participants

This single-center, retrospective, and observational study analyzed 316 critically ill COVID-19 patients in the ICU of Jinyintan Hospital in Wuhan, China from February 1, 2020, to March 20, 2020. All patients met the diagnostic criteria according to the World Health Organization interim guidance [14]. Critically ill patients were defined as those admitted to the ICU who required mechanical ventilation, or shock, or combined with other organ failure requires ICU care [15]. There were 260 critically ill patients who received at least standard doses of pharmacologic thromboprophylaxis (low-molecular-weight heparin, LMWH, subcutaneous injection) for more than 1 week after ICU admission according to their venous thromboembolism risk assessment and bleeding risk assessment [3, 16]. A total of 172 patients were excluded due to no surveillance results on DVT or pharmacologic thromboprophylaxis before ICU admission. Finally, 88 patients got the surveillance of lower limb compression ultrasonography (CUS) (LOGIQ e, GE) for deep venous thrombosis after LMWH thromboprophylaxis. The deep veins below the knee, including peroneal veins, posterior tibial veins, anterior tibial veins, and gastrocnemius muscle veins were defined as “distal” deep veins [17]. Those veins, such as greater saphenous veins, popliteal veins, and femoral veins were called as “proximal” deep veins. Every patient received echocardiography scan, especially for identifying pulmonary arterial hypertension.This study was approved by Jinyintan Hospital Ethics Committee (KY-2020-06.01). Written informed consent was waived by the Ethics Committee due to the emergence of this infectious disease.

### Data collection

A trained team of physicians retrospectively reviewed clinical electronic medical records and laboratory findings for all the patients. We collected data on age, sex, the dates of disease onset, ICU admission, and discharge or death, the length of ICU stay, chronic disease history (hypertension, diabetes, hematencephalon, cerebral infarction, malignancy, gastric ulcer, thyroid disease, hepatitis B, fatty liver, etc.) and bleeding events. The laboratory values (complete blood count, coagulation profile, serum biochemical tests, PaO_2_/FiO_2_ ratio, etc.) and transthoracic echocardiogram reports nearest to the time of lower limb CUS examination were collected. Risk assessment models of DVT (Padua score, IMPROVE score) [16] and Sequential Organ Failure Assessment (SOFA score) [18] were calculated retrospectively according to the medical records by a blinded reviewer (critical care fellow).

### Outcome

The main outcomes were the incidence of DVT (distal and proximal DVT), risk factors of DVT, and bleeding events in critically ill COVID-19 patients with pharmacological prevention. The secondary outcome of interest was in-hospital mortality.

### Statistical analysis

Descriptive data were expressed as mean (SD) or median (IQR) for continuous variables and number (%) for categorical variables. Comparison of continuous variables was examined by independent Student’s t-test or Mann–Whitney U test. Statistical analysis of categorical variable was performed using the Pearson chi-square test and Fisher’s exact test as appropriate. Firth logistic regression was used to estimate odds ratio (OR) with 95% confidence interval (CI) to evaluate the association of the incidence of DVT with demographic and clinical characteristics. All statistical analyses were performed using SAS 9.4.

## Results

**Fig. 1:**
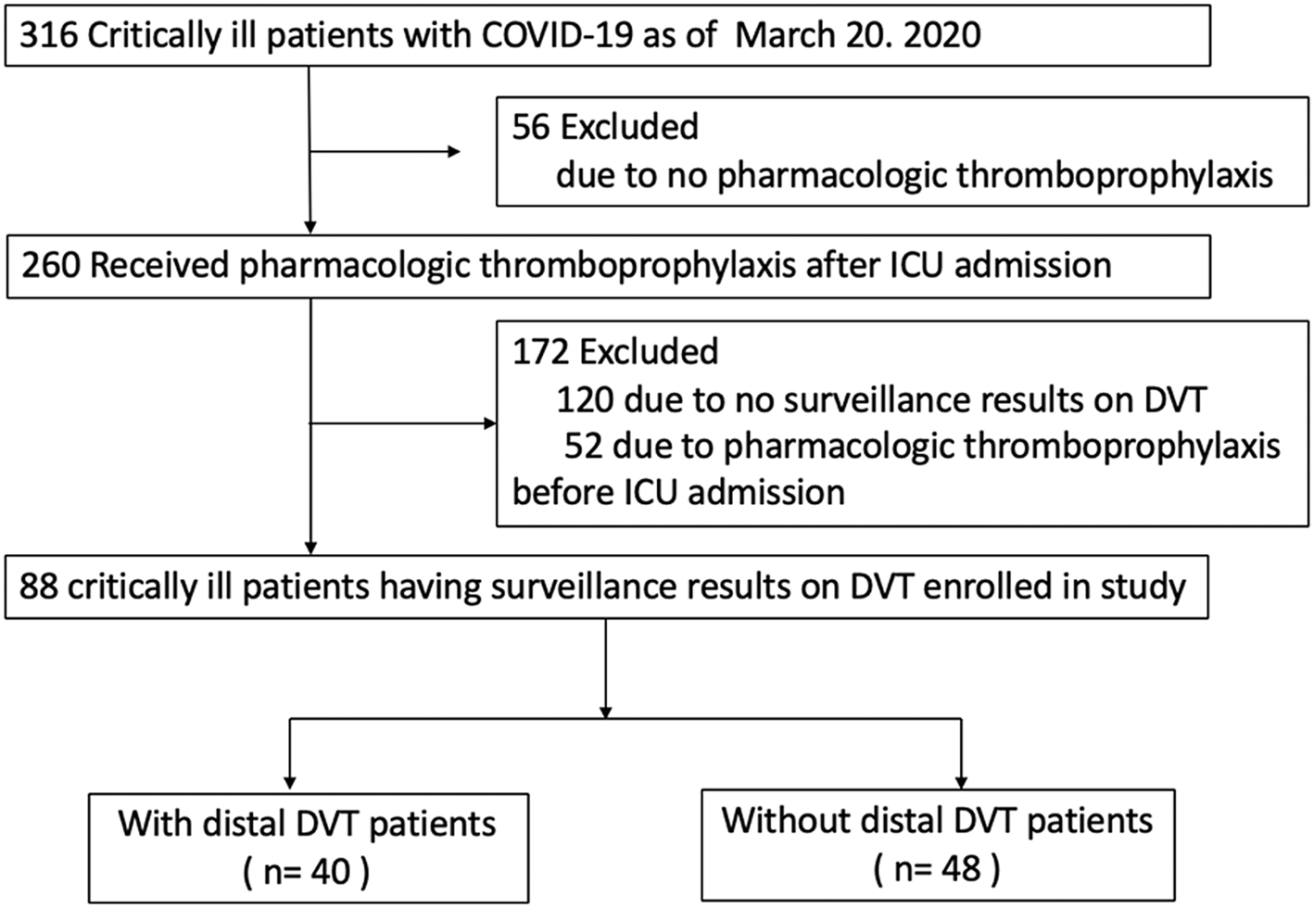
Flowchart of the study population

We retrospectively enrolled 88 identified patients in the ICU with critically ill COVID-19 (Fig. 1). All of the patients had VTE pharmacologic thromboprophylaxis prescribed LMWH for more than 1 week, which was appropriate and consistent with current institutional and national guidelines [19]. The median (interquartile range [IQR]) age was 63 (55–71) years old and 34 (39%) were female (Table 1). Upon study entry, the median (IQR) SOFA score was 5 (4–6). The SOFA score of DVT patients was significantly higher than that of non-DVT patients (6 [5–7] vs 4 [3–5], *P* < 0.001). In the ICU, 74% of patients required mechanical ventilation, and 26% received high-flow nasal cannula (HFNC), respectively.

**Table 1:** Demographics and clinical characteristics of critically ill COVID-19 patients with or without distal DVT (dDVT)

	All patients (N = 88)	Patients with dDVT (N = 40)	Patients without dDVT (N = 48)	*P* value
Age (years)	63 (55, 71)	63 (56, 70)	64 (55, 73)	0.914
Sex				
Male	54 (61%)	25 (63%)	29 (60%)	0.842
Female	34 (39%)	15 (38%)	19 (40%)	
PaO_2_/FiO_2_ ratio	150 (100, 199)	121 (95, 190)	156 (109, 201)	0.093
SOFA score	5 (4,6)	6 (5,7)	4 (3,5)	< 0.001
Required respiratory support				
HFNC	23 (26%)	8 (20%)	15 (31%)	0.191
Noninvasive ventilation	32 (36%)	13 (33%)	19 (40%)	
Invasive mechanical ventilation	33 (38%)	19 (48%)	14 (30%)	
Laboratory parameters				
Leukocyte, × 10^9^/L	7.90 (5.79, 12.36)	7.07 (4.93, 12.11)	8.51 (6.71, 12.87)	0.054
Neutrophils, × 10^9^/L	6.44 (4.49, 10.74)	5.95 (3.48, 10.74)	6.89 (4.92, 10.52)	0.164
Lymphocytes, × 10^9^/L	0.78 (0.56, 1.12)	0.75 (0.60, 1.04)	0.84 (0.53, 1.20)	0.527
Platelets, × 10^9^/L	197 (139.50, 277)	183 (123.50, 221.50)	217.50 (147.50, 314.50)	0.037
Haemoglobin (g/L)^a^	119 (106, 136)	125 (112.50, 138.50)	114 (96, 128)	0.015
D-dimer (mg/L)	4.35 (1.99, 10.10)	6.41 (2.75, 10.94)	3.10 (1.39, 7.60)	0.020
BNP (pg/mL)	94.85 (37, 229)	108 (33.50, 218.50)	82.75 (50, 256)	0.466
CRP (mg/L)	40.20 (6.74, 74.70)	44.75 (5.55, 74.75)	30.10 (8.35, 74.45)	0.860
PCT (ng/mL)	0.13 (0.05, 0.16)	0.14 (0.13, 0.23)	0.05 (0.05, 0.05)	< .001
ESR (mm/H)	43.57 (24.90)	32.92 (22.40)	53 (23.34)	< .001
Cr (µmol/L)	68.75 (58.20, 87.65)	70.90 (59.15, 89.15)	67.70 (56.10, 82.60)	0.988
BUN (mmol/L)	4.20 (3.21, 5.79)	4 (3.17, 6.30)	4.25 (3.30, 5.10)	0.373
LDH (U/L)	288.50 (212, 365)	288.50 (206, 361)	291 (224, 415)	0.586
cTnI (ng/ml)	5.25 (3, 11.10)	4.40 (2.60, 9.65)	5.50 (3.70, 11.55)	0.202
PT (s)	13.10 (12.60, 13.80)	12.90 (12.60, 13.60)	13.20 (12.65, 14.10)	0.210
APTT (s)	38.65 (36.15, 41.65)	39.15 (36.25, 42.75)	38.25 (35.85, 40.60)	0.227
Albumin (g/L)	30.76 (4.16)	28.81 (3.91)	32.39 (3.67)	< .001
Medical history				
Hypertension	31 (35%)	12 (30%)	19 (40%)	0.349
Diabetes	9 (10%)	6 (15%)	3 (6%)	0.290
Hematencephalon	2 (2%)	0 (0%)	2 (4%)	0.498
Cerebral infarction	3 (3%)	1 (3%)	2 (4%)	1.000
Malignancy	5 (6%)	0 (0%)	5 (10%)	0.061
Gastric ulcer	1 (1%)	1 (3%)	0 (0%)	0.455
Thyroid diseases	1 (1%)	0 (0%)	1 (2%)	1.000
Coronary heart disease	2 (2%)	2 (5%)	0 (0%)	0.204
Hepatitis B	1 (1%)	0 (0%)	1 (2%)	1.000
Fatty liver	2 (2%)	1 (3%)	1 (2%)	1.000
Pulmonary arterial hypertension, n (%)	36 (41%)	21 (53%)	15 (31%)	0.044
Time from disease onset to dDVT confirmed (days)	28 (16, 40)	34 (22, 42)	20 (15, 32)	0.007
Time from ICU admission to dDVT confirmed, days	9 (7, 11)	10 (8, 12)	8 (7, 11)	0.067
ICU length of stay, days	22 (18, 30)	27 (19, 32)	21 (18, 29)	0.257
Padua score				
< 4	12 (14%)	0 (0%)	12 (25%)	< .001
≥ 4	76 (86%)	40 (100%)	36 (75%)	
Wells score				
≤ 0	12 (14%)	0 (0%)	12 (25%)	
1–2	68 (77%)	33 (83%)	35 (73%)	< .001
≥ 3	8 (9%)	7 (17%)	1 (2%)	
IMPROVE VTE RAM				
2–3	80 (91%)	40 (100%)	40 (83%)	0.007
≥ 4	8 (9%)	0 (0%)	8 (17%)	
IMPROVE bleeding RAM				
< 7	61 (69%)	32 (80%)	29 (60%)	0.047
≥ 7	27 (31%)	8 (20%)	19 (40%)	
Bleeding adverse event				
No	83 (94%)	38 (95%)	45 (94%)	1.000
Yes	5 (6%)	2 (5%)	3 (6%)	
Mortality	20 (23%)	12 (30%)	8 (17%)	0.137

*DVT* deep vein thrombosis, *SOFA* sequential organ failure assessment, *HFNC* high-flow nasal cannula, *BNP* brain natriuretic peptide, *CRP* C-reactive protein, *PCT* procalcitionin

Although more patients with distal DVT suffered lower arterial partial pressure of oxygen and required invasive mechanical ventilation than those without distal DVT, there was no statistically significant difference between them. Patients with DVT had a significantly longer disease duration (days from disease onset to CUS performed) compared with patients without DVT (34 [22–42] vs 20 [15–32] days, *P* = 0.007). ICU length of stay in patients with DVT did not differ from patients without DVT (27 [19–32] days vs 21 [18–29] days, *P* = 0.257). Incidence of pulmonary arterial hypertension that occurred in patients with DVT was significantly higher than in patients without DVT (53% vs 32%, *P* = 0.044). ARDS occurred in 81% (31 of 40) of patients with DVT, and 70% (33 of 48) of patients without DVT (*P* = 0.191). All-cause, 28-day mortality was numerically higher in patients with DVT but did not reach statistical significance (30% vs 17%, *P* = 0.137). All bleeding complications (5/88) were non-fatal.

### Incidence of DVT

Acute DVT (proximal and/or distal DVT) occurred in 40 patients (46%, 95% CI 35%-56%). D-dimer of those patients was all positive and mean level was 4.35 mg/L (95%CI, 1.99–10.1) at the time CUS performed (Table 1). Proximal DVT was recognized in 9.1% (eight of 88) of patients, and distal DVT in 46% (40 of 88) of patients (Table 2). All of the proximal DVT combined with distal DVT. The distal DVT patients all had risk factors of DVT extension (such as positive D-dimer, no reversible provoking factor for DVT, and inpatient status) [19]. The majority of proximal DVT patients (63%, five of eight) and distal DVT patients (80%, 32 of 40) were asymptomatic. Gastrocnemius muscle veins were the most likely site where thrombosis occurred (80%, 32 of 40]). Bilateral distal DVT occurred in 65% (26 of 40) of DVT patients. DVT occurred in 68% (26 of 38) of patients with hypoalbuminemia (< 30 g/L) and 28% (14 of 50) of patients without hypoalbuminemia.

**Table 2:** Summary of the imaging for DVT by lower limb compression ultrasonography

	Cases, n	%	Left side	Right side
Unilateral distal DVT	14	35		
Left calf	5	36		
Right calf	9	64		
Posterior tibial veins			3	3
Anterior tibial veins			1	2
Peroneal veins			2	3
Gastrocnemius muscle veins			3	7
Bilateral distal DVT	26	65		
Posterior tibial veins	15	59		
Anterior tibial veins	5	19		
Peroneal veins	12	46		
Gastrocnemius muscle veins	22	85		
Proximal DVT	8			
Greater saphenous vein	2			
Popliteal vein	6			
Superficial femoral vein	14			

Incidence of DVT in patients with Padua score ≥ 4 was significantly higher than patients with Padua score < 4 (53% vs 0%, *P* < 0.001). Similarly, 50% (40 of 80) of patients with IMPROVE score 2–3 developed DVT, however, no patients with IMPROVE score ≥ 4 developed DVT. The incidence of DVT in patients with Wells score 1–2 was significantly lower than patients with Wells score ≥ 3 (49% vs 88%, *P* = 0.040).

### Risk factors of DVT

Univariate regression analyses found that hypoalbuminemia, illness severity (as determined by SOFA score), and disease duration (time from disease onset to distal DVT confirmed) were independently associated with DVT (Table 3). Active cancer was not considered for analyses because of the small number of patients with cancer (n = 4) in our cohort. We explored other potential predictors, including BMI, age, sex, PaO_2_/FiO_2_ ratio, and platelet counts, but none of these was significantly associated with DVT in univariate analyses.

**Table 3:** Association of distal DVT with demographic and clinical characteristics

Risk factors	Univariate OR	*P* value	Multivariate OR	*P* value
Age (per IQR [5])	1.01 (0.86, 1.18)	0.920	1.00 (0.81, 1.24)	0.993
Gender (male)	1.09 (0.46, 2.57)	0.849		
SOFA score	1.87 (1.39, 0.64)	0.000	2.07 (1.38, 3.39)	0.001
D-dimer (per IQR [8])	1.20 (0.95, 1.52)	0.123	1.38 (1.03, 1.84)	0.029
Hypoalbuminemia (per IQR [6])	0.24 (0.11, 0.52)	0.000	0.17 (0.06, 0.05)	0.001
PaO_2_/FiO_2_ ratio (> 150)	0.54 (0.23, 1.23)	0.150		
Required invasive mechanical ventilation	2.16 (0.91, 5.22)	0.086		
Time from disease onset to distal DVT confirmed	1.04 (1.01, 1.07)	0.017	1.04 (1.00, 1.08)	0.084

In multivariable analyses, three factors remained as significant DVT risk factors: hypoalbuminemia (OR per IQR 0.17; 95% CI 0.06–0.05, *P* = 0.001), SOFA score (OR 2.07; 95% CI 1.38–3.39, *P* = 0.001), and D-dimer (OR per IQR 1.04; 95% CI 1.03–1.84, *P* = 0.029) (Table 3). Time from disease onset to distal DVT confirmed did not reach statistical significance (OR 1.04; 95%CI 1.00–1.08, *P* = 0.084) in multivariable analyses.

## Discussion

In this study, we investigated the DVT incidence, outcomes, and risk factors in patients admitted to the ICU with critically ill COVID-19 after LMWH thromboprophylaxis. Our study indicated that incidence of DVT was high (46%) in patients with critically ill COVID-19 despite the use of universal, guideline-recommended thromboprophylaxis. Bilateral distal DVT was more common than unilateral distal DVT. The majority of patients with distal DVT were asymptomatic, and gastrocnemius muscle veins were the most likely site where thrombosis occurred.

In our study, the majority of DVT patients had distal DVT, which all had risk factors of DVT extension and were suggested to prescribe anticoagulants over serial imaging of the deep veins according to AACP guideline [19]. The risk factors for extension of distal DVT included positive D-dimer, extensive thrombosis, thrombosis closed to the proximal veins, no reversible provoking factor for DVT, active cancer, DVT history, and inpatient status [19]. Actually, all these distal DVT patients in our study had at least three risk factors, such as positive D-dimer, no reversible provoking factor for DVT, and inpatients status. From our results, pulmonary hypertension in patients with distal DVT more likely occurred than in patients without distal DVT, which probably be induced by pulmonary embolism. Our findings highlighted that currently recommended VTE prophylaxis and management strategies may not be suitable in severe COVID-19 compared with other critically ill populations. Therefore, we suggested that all these patients with whether proximal or distal DVT should be adjusted the treatment strategy from prophylactic anticoagulation to therapeutic anticoagulation. As such, randomized controlled trials specifically studying thromboprophylaxis in patients with severe COVID-19 are necessary to improve the prevention of VTE in this critical illness.

Moreover, our study recognizied that critically ill COVID-19 patients had a markedly high incidence of DVT. Some studies indicated different incidence of DVT in critically ill COVID-19 patients varied greatly in different sites. For example, a retrospective study of 81 critically ill COVID-19 patients from China reported that DVT occurred in 25% of these patients without thromboprophylaxis [12]. However, retrospective study from France found that the incidence of DVT was 69% (18 of 26) with thromboprophylaxis [13]. Another retrospective study from Netherlands indicated that the incidence of DVT was 32% (24 of 75) with thromboprophylaxis [20]. Our findings were similar with these studies from Europe.

In addition, in a multicenter prospective study, incidence of DVT was 34% in patients with severe sepsis and septic shock, which was close but lower to our findings [21]. Compared with other patients in ICU without seriously infectious illness, DVT occurred in majority of patients with critically ill COVID-19 [21]. Our study also indicated that hypercoagulability in critical COVID-19 differed from that of other infectious critical illnesses. Although exact mechanisms were still unclear, COVID-19 led to coagulation dysfuntion with markedly elevated D-dimer and fibrinogen degradation products (FDP) [22]. These factors induced hypercoagulability and might partly explain the high incidence of DVT in critically ill COVID-19 patients in the our study.

Furthermore, we found that the presence of hypoalbuminemia, SOFA score, and elevated D-dimer were predictors of DVT. Hypoalbuminemia is associated with highly increased DVT risk (almost twofold higher risk), which builds on studies in acutely ill patients, and which we now identify as being a risk factor in critical COVID-19 as well. Several anticoagulative properties of albumin have been proposed to explain the relationship between low albumin and increased VTE risks, which include inhibiting fibrin polymerization and platelet aggregation, enhancing the effect of antithrombin III, and promoting hepatic synthesis of coagulation factors [23]. These findings highlight the importance of sustain normal albumin level in critical COVID-19. The VTE incidence of COVID-19 was higher in the ICU than on the wards [20]. Similarly, we found that higher SOFA score markedly increased DVT risk. High D-dimer was also a significant risk factor of DVT. Hence, when the levels of D-dimer are non-proportionally high, DVT should be considered.

The strengths of our study included its comprehensive data collection, our tracking of the type and duration of thromboprophylaxis, and details of proximal and distal DVT. As the SOFA score, PaO_2_/FiO_2_ ratio and ARDS incident were different in DVT group and non-DVT group, it indicated that patients with DVT were more serious than those without DVT. These may be the reason that mortality was higher in patients with DVT compared with those without DVT, although it did not reach statistical significance. Our data showed that COVID-19 related DVT hindered clinical care and worsed patient outcomes, although studies with larger sample were needed to further estimate the clinical effect of COVID-19 related DVT. Recognizing DVT in patients with COVID-19 was anticipated to pose a significant challenge, as the signs and symptoms of DVT might be abscent in majority of patients. Although compression ultrasonography screening for DVT in patients without symptoms has not been suggusted, clinicians should screen most of critically ill COVID-19 patients for DVT.

Our study had some limitations. Firstly, we did not include thrombophilia, prior VTE and other VTE risk factors. And the sample size was too small. As we just performed a retrospective study, the high incidence of DVT was needed to be indentified by larger prospective trials in critical ill COVID-19 patients. In addition, DVT which occurred before ICU admission cannot be excluded absolutely. Finally, these patients were not received CTPA for diagnosis of pulmonary embolism as CT scans were not always easy to perform due to critically ill COVID-19 patients positioning in ICU.

## Conclusion

Patients with critically ill COVID-19 had a high incidence of DVT, despite the use of universal, guideline-recommended pharmacologic thromboprophylaxis. The presence of hypoalbuminemia, higher SOFA score, and elevated D-dimer were independent risk factors of DVT. More effective VTE prevention strategies are necessary for patients with COVID-19, and future researches in this population are urgently needed.
